# FDG PET-CT for the Detection of Occult Nodal Metastases in Head and Neck Cancer: A Systematic Review and Meta-Analysis

**DOI:** 10.3390/cancers16172954

**Published:** 2024-08-24

**Authors:** Danaé Guedj, Sophie Neveü, Minerva Becker, Maxime Mermod

**Affiliations:** 1 Clinic of Otorhinolaryngology-Head and Neck Surgery, Department of Clinical Neurosciences, Geneva University Hospitals (HUG), 1205 Geneva, Switzerland; 2 Department of Pathology and Immunology, University of Geneva (UNIGE), 1206 Geneva, Switzerland; 3 Division of Radiology, Unit of Head and Neck and Maxillo-Facial Radiology, Diagnostic Department, Geneva University Hospitals (HUG), 1205 Geneva, Switzerland; sophie.neveu@hug.ch (S.N.); minerva.becker@hug.ch (M.B.)

**Keywords:** FDG PET-CT, occult metastasis, head and neck squamous cell carcinoma, clinically node-negative neck (cN0)

## Abstract

**Simple Summary:**

With the aim of offering precision medicine to our patients, accurate lymph node staging of head and neck (HN) cancer patients is a major challenge, as it determines whether neck dissection is indicated. For many years, the indication for this surgery has been histological predictors from the primary tumor only, and efforts are converging to develop non-invasive techniques to exclude occult metastases with certainty. FDG PET-CT plays a dominant role in this respect. The aim of our systematic review and meta-analysis was to determine the overall diagnostic performance of FDG PET-CT for the detection of LN metastases from HN squamous cell carcinoma (HNSCC) in patients with clinically node-negative necks.

**Abstract:**

Because of an estimated 20–30% prevalence of occult lymph node (LN) metastases in patients with head and neck squamous cell carcinoma (HNSCC), neck dissection is often proposed, despite its potential morbidity. In this systematic review and meta-analysis, the diagnostic performance of FDG PET-CT in detecting occult LN metastases was evaluated in patients with clinically negative necks (cN0) and in whom histopathology of a neck dissection specimen served as gold standard. Overall, 16 studies out of 2062 screened on PubMed and EMBASE fulfilled the inclusion criteria (n = 1148 patients). Seven of these sixteen studies were split into two or three studies because they contained data that could be processed distinctly in our meta-analysis. For this reason, a total of 25 studies were identified and included in the analysis (n total = 1918 patients). The overall prevalence of metastatic nodes per patient was 22.67%. The pooled sensitivity, specificity, diagnostic odds ratios, and negative predictive value (NPV) were 0.71 (95%CI: 0.66–0.75), 0.90 (95%CI: 0.84–0.93), 20.03 (95%CI: 13.51–29.70), and 0.92 (95%CI: 0.89–0.95), respectively. The main causes of inter-study heterogeneity included different reference standards (evaluation per patient, per neck side, or per neck level). The current meta-analysis showed that FDG PET-CT has a high specificity and NPV for ruling out nodal involvement in cN0 necks, but a limited sensitivity.

## 1. Introduction

Head and neck squamous cell carcinoma (HNSCC) is the sixth most common cancer worldwide and accounts for approximately 900,000 new cancer cases each year [1]. Regional metastasis of HNSCC is the most important prognostic factor for overall survival, and the presence of a single lymph node metastasis (LNM) in the neck is already associated with a 50% drop in survival [2]. Occult LNMs (OLNMs) correspond to tumor cell deposits in lymph nodes of patients considered free of disease in the neck after clinical and radiological examinations, so-called clinical N0 necks (cN0). OLNMs occur in 20% to 30% of patients with HNSCC regardless of their primary tumor (T) classification [3]. Until recently, elective neck dissection, watchful waiting, and sentinel lymph node biopsy (SLNB) were the main management strategies for the cN0 neck. Prospective data published by D’Cruz et al. showed that elective neck dissections were associated with a survival benefit compared to watchful waiting for patients with oral SCC with a depth of invasion superior to 3 mm [4]. SLNB represents an attractive alternative that is oncologically equivalent to elective neck dissection [5] and has less morbidity [6]. However, SLNB still requires surgical intervention under general anesthesia and carries the risks of any invasive procedure. Several investigators have tried to overcome those limitations by identifying histologic parameters or genetic signatures predicting OLNM [7,8,9]. However, no parameter, except depth of tumor infiltration, has undergone prospective clinical validation and made it to clinical practice so far.

To date, no imaging technique is reliable enough to replace invasive neck treatment. In practice, every patient presenting with HNSCC undergoes palpation and imaging of the neck, with modality selection based on the location of the primary tumor and the presence or absence of cervical adenopathy on palpation. FDG PET-CT is currently reserved for the staging of advanced disease and typically not for the detection of OLNM in early disease. Interestingly, PET-CT may have a higher accuracy than MRI and CT in this clinical context. This gap in applicability highlights the need for further investigation into the role of PET-CT in this context. Several researchers have investigated the diagnostic performance of FDG PET-CT for detecting OLNMs, albeit obtaining very heterogeneous results. This study systematically reviews the diagnostic accuracy of various imaging modalities, including CT, MRI, and PET-CT, for detecting cervical lymph node metastasis in head and neck cancer. PET-CT was found to have the highest diagnostic accuracy among the modalities studied, particularly in detecting metastatic lymph nodes.

The present systematic review and meta-analysis aimed to assess the diagnostic performance of FDG PET-CT in detecting OLNM in HNSCC patients with clinically or clinico-radiologically cN0 necks.

## 2. Materials and Methods

This study was conducted following the PRISMA statement [10] and the Cochrane handbook of systematic reviews of interventions [11]. No ethics committee approval was required for this meta-analysis. The meta-analysis was not registered in PROSPERO.

### 2.1. Database Search and Selection Strategy

MEDLINE and EMBASE databases were systematically screened for articles published between January 2000 and July 2024. The non-exhaustive list included the following keywords: “Head and Neck Squamous Cell Carcinoma (HSNCC)”, “cervical metastasis”, and “Positron-Emission-Tomography” combined using AND/OR operators. Synonymous terms were added according to their definition in the database. The term NOT was explicitly avoided to reduce the risk of excluding relevant articles. The references of relevant systematic reviews/meta-analyses were searched to identify additional potential studies. Full details of the search strategies are indicated in Appendix A and Appendix B.

The eligibility criteria for inclusion in the current meta-analysis were as follows:

(1) HNSCC was defined as squamous cell carcinoma in the oral cavity, oropharynx, hypopharynx, and larynx. Locations such as unknown primary, nasopharynx, salivary glands, head and neck skin, paranasal sinuses, and ear were excluded.
(2) Primary HNSCC proven histologically.
(3) No synchronous oncological disease.
(4) Clinically N0 neck (cN0).
(5) No prior oncologic treatment.
(6) Histological analysis of a neck dissection specimen used as a gold standard.
(7) PET combined with both non-contrast-enhanced (NCE) or contrast-enhanced CT (CECT).
(8) PET-CT acquisitions after the year 2000.
(9) Available or retrievable data for true positive (TP), true negative (TN), false positive (FP), and false negative (FN) evaluations.

**Figure 1:**
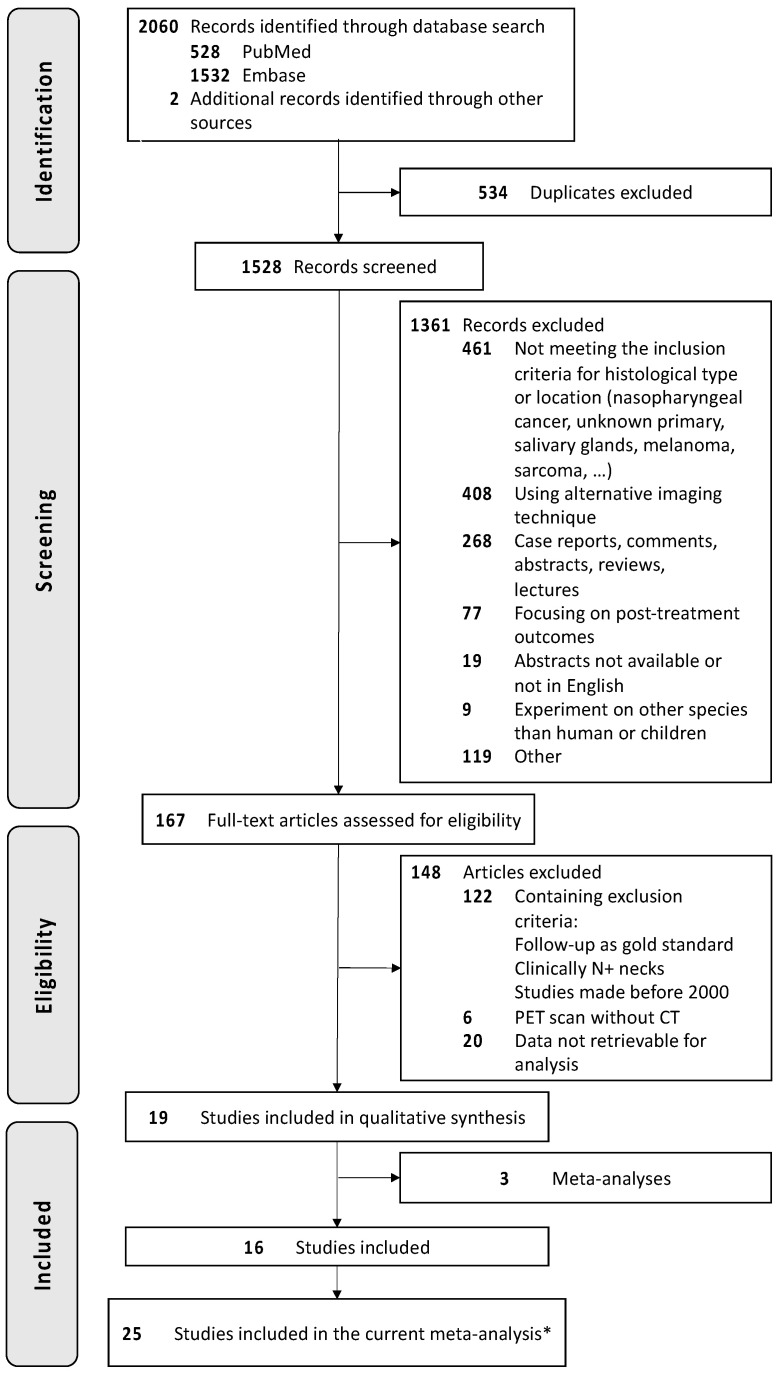
The PRISMA flow-chart of study selection. * Considering the various results available within a single study.

The study strictly followed the PRISMA (Preferred Reporting Items for Systematic Reviews and Meta-Analyses) guidelines, and the PRISMA search flow diagram is provided in Figure 1.

### 2.2. Data Extraction

A standard data extraction was performed and documented in a Microsoft Excel file. All useful study characteristics were referenced, including year of publication, study design, number of patients included, TP, TN, FP, FN, sensitivity, specificity, accuracy, positive predictive value (PPV), and negative predictive value (NPV) for the purpose of statistical analysis. The QUADAS-2 score was also calculated for each study. Study selection and data extraction were performed independently by two investigators. Discrepancies regarding the extracted studies were resolved by consensus. As the majority of studies have defined cN0 solely on the basis of palpation, we decided in this study to distinguish between cN0 necks described exclusively by clinical examination and cN0 necks defined by clinical examination and one or more other imaging techniques (clinico-radiological) as defined in the TNM 8th edition [12].

### 2.3. Data Analysis

In diagnostic test studies, sensitivity and specificity are often correlated. Therefore, pooling them in a meta-analysis may lead to biased results. Reitsma et al. proposed a bivariate approach [13], whereas alternative approaches include the hierarchical summary (HS) Receiver Operating Characteristic curve (ROC) model [14]. Both approaches lead to similar results [15]. The current study used the Reitsma bivariate approach implemented in the Mada R package [16]. A random effect model was applied to account for the differences between studies’ sensitivity. Variance components were estimated by restricted maximum likelihood (REML). The bivariate analysis results are displayed as summary ROC (SROC). Since the Mada R package does not provide the total effect sizes for summary statistics but only provides the effect size of individual studies as a forest plot, the value of each summary statistic was calculated by performing univariate analysis using the meta R package [17]. To investigate the presence of publication biases, Deeks funnel plots [18] were obtained based on a weighted linear regression of the log diagnostic odds ratio (DOR) on the inverse of the square effective sample size using the adequate sample size as weights. This test for funnel plot asymmetry is also a function in the meta R package [17]. Finally, an influence analysis was performed to account for the stability of the detected results. To do so, a leave-one-out analysis investigating the influence of each individual study on the global effect size was conducted. The effect of potentially confounding variables (type of study, sample size, year of publication, definition of cN0, type of histological processing, reference standard, localization) was investigated by adding moderator variables to the bivariate regression model. Variables that had a statistically significant impact on the results were then used to create subgroups subject to separate meta-analyses. All computations were performed using the R statistical programming language version 4.0.3 (2020-10-10) [19].

## 3. Results

### 3.1. Systematic Review

#### 3.1.1. Search Results

The search strategy yielded 2062 studies, and citation tracking added 2 additional studies. After duplicates were removed, 1528 titles and abstracts were screened, and 167 full papers were assessed for eligibility. Finally, 19 studies fulfilled our inclusion criteria, and 3 of them were excluded because they were meta-analyses. Of the sixteen studies included in our meta-analysis, ten were prospective, and six were retrospective (Table 1).

In Lee et al. [25], Sohn et al. [26], and Kanamura et al. [33], the diagnostic performance of FDG PET-CT was calculated using a per patient as well as a per neck level analysis. In Schöder et al. [20], the evaluation of the diagnostic performance was per neck side and per neck level; in Piao et al. [21], the results were provided per neck level and per lymph node. In Roh et al. [24] and Bae et al. [30], results were per patient, neck side, and neck level.

We decided to consider each given reference standard as a single study (although based on the same sample). Therefore, the final sample consisted of 25 studies with a total of 1918 patients.

Regarding the definition of cN0, sixteen studies (n = 1324 patients) defined cN0 based only on negative neck palpation or the authors did not mention whether cross-sectional imaging was also used to classify necks as cN0. Nine studies (n = 596 patients) used clinical evaluation combined with cross-sectional imaging techniques for the definition of cN0. Among these nine studies, the authors used clinical evaluation combined with a mix of negative CT, MRI, or ultrasound results to define cN0 necks in eight studies, whereas one study used clinical evaluation and CT only.

#### 3.1.2. Study Characteristics and Quality Assessment

**Figure 2:**
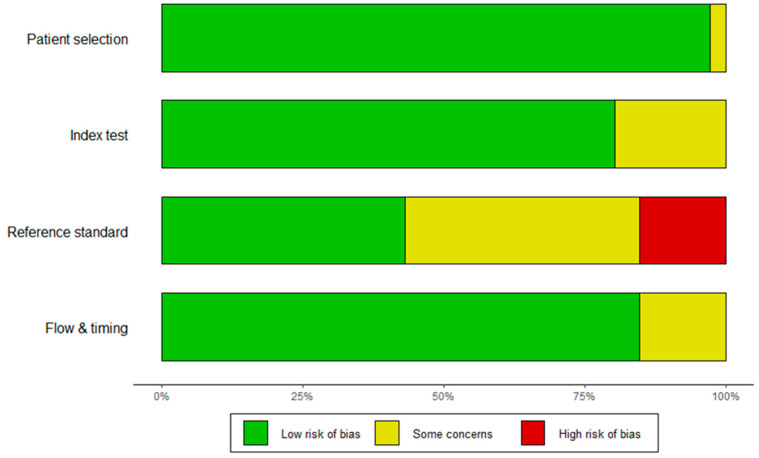
Risk of bias graphical representation using sample size as a weighting method.

Study quality was evaluated using the quality assessment of diagnostic accuracy studies (QUADAS-2) tool, which is recommended for the evaluation of the risk of bias and applicability in diagnostic accuracy studies. The assessment tool focuses on four key domains: patient selection, index test, reference standard, and flow and timing. The risk of bias was evaluated in all four domains, and applicability concerns were evaluated in the first three domains. For each question, the risk of each study was graded as “low”, “high”, or “some concerns”. A standardized table, recommended by the QUADAS-2 official website, was used to display the summarized results of the study quality appraisal (Figure 2).

#### 3.1.3. Publication Bias and Sensitivity Analysis

**Figure 3:**
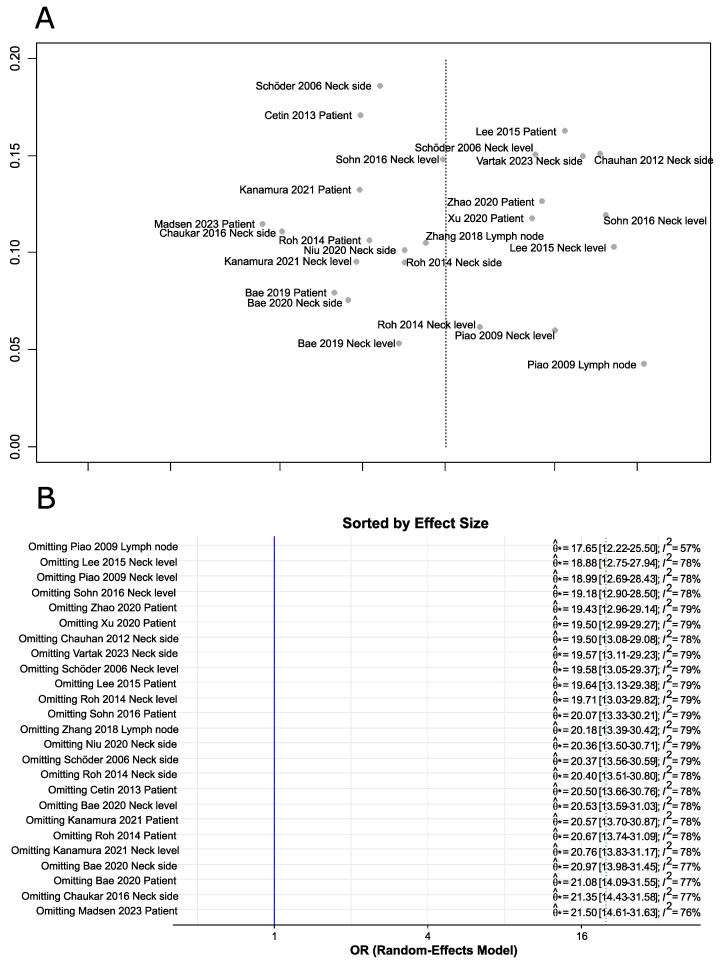
Results of influence analysis. (**A**) Funnel plot test for diagnostic odds ratios. (**B**) Leave-one-out analysis sorted by effect size [20,21,22,23,24,25,26,27,28,29,30,31,32,33,34,35].

The shape of the funnel plots revealed no asymmetry (Figure 3A). To test for forest plot asymmetry, the test statistics proposed by Deeks et al. [18] based on a weighted linear regression of the log diagnostic odds ratio on the inverse of the square effective sample size using the effective sample size as weights was performed. This test confirmed that no significant asymmetry could be detected (*p*-value = 0.14). The study by Piao et al. [21] with lymph node as a denominator was particularly influential in the meta-analysis, as omitting this study significantly changed the effect size and reduced heterogeneity (Figure 3B).

#### 3.1.4. Pooled Analysis of the Diagnostic Performance of PET-CT for the Detection of Occult Lymph Node Metastasis (OLNM)

The diagnostic performance was reported in all 25 studies, comprising 1918 patients. Prevalence of OLMNM could only be computed for the subgroup of studies using the patient as a denominator. In this subgroup, the prevalence of OLNM was 22.67%. The pooled sensitivity, specificity, and diagnostic odds ratio (DOR) were 0.71 (95%CI: 0.66–0.75), 0.90 (95%CI: 0.84–0.93), and 20.03 (95%CI: 13.51–29.70), respectively (Table 1). The NPV was 0.92 (95%CI: 0.89–0.95). Heterogeneity between studies was high. The I^2^ statistic for sensitivity, specificity, DOR, and NPV was 0.38 (95%CI: 0.0–0.61), 0.95 (95%CI: 0.93–0.96), 0.78 (95%CI: 0.68–0.85), and 0.90 (95%CI: 0.85–0.92), respectively.

#### 3.1.5. Meta-Regression Analysis Reveals Sources of Heterogeneity

Because heterogeneity between studies was high, bivariate meta-regression was performed to explore sources of heterogeneity (Table 2).

The type of denominator was the only significant source of heterogeneity. Neck side vs. other categories was associated with a statistically significant change in sensitivity and false positive rate (*p*-value = 0.03 and *p*-value = 0.001, respectively). The test’s sensitivity was higher when the denominator was “Neck side” or “Patient” compared to the reference category, indicating that the test was more effective at correctly identifying true positives in these groups. Conversely, the test’s false positive rate was higher when the denominator was “Neck level”, “Neck side”, or “Patient” compared to the reference category, suggesting a greater likelihood of incorrectly identifying healthy individuals as having the condition in these groups.

In summary, using “Neck side” or “Patient” as the denominator enhanced the test’s ability to correctly identify true positives but also increased the likelihood of false positives.

The type of study was a source of heterogeneity as the false positive rate decreased significantly for retrospective studies, dropping from 14.8% in prospective studies to about 8.5% in retrospective studies. This effect disappeared after omitting the single most influential study of Piao et al. [21].

**Figure 4:**
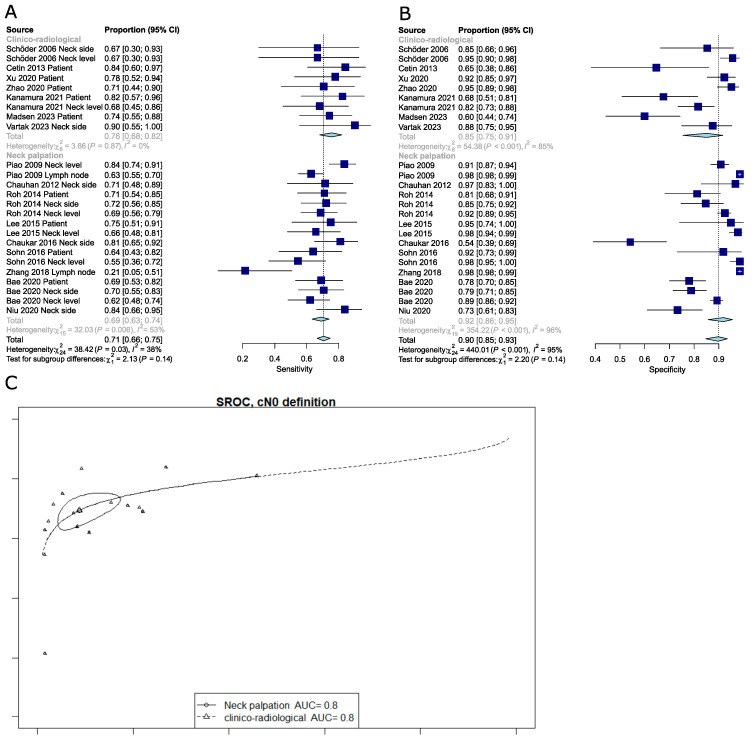
(**A**) Forest plot of sensitivity for different definitions of cN0. (**B**) Forest plot of specificity for different definitions of cN0. (**C**) SROC curve for different definitions of cN0 [20,21,22,23,24,25,26,27,28,29,30,31,32,33,34,35].

The technique used to determine that a neck is cN0 (clinical examination and palpation vs. clinical examination combined with US, MRI, or CT) was not a source of heterogeneity. High variance within subgroups could have obscured actual differences between subgroups by increasing the overall noise in the data, making it more challenging to detect significant differences between subgroup means. This increased within-subgroup variance reduces the statistical power of tests and complicates the identification of meaningful differences between the groups under study (Figure 4).

When the standard of reference was histology without serial slice analysis, PET-CT had a sensitivity of approximately 70.7% in correctly identifying patients with positive nodes. Niu et al. [29], Schöder et al. [20], and Zhao et al. [32] used a serial histologic evaluation technique for LNs; the use of a serial technique marginally increased the sensitivity to about 74.3%. However, this increase was not statistically significant.

Year of publication, sample size, and localization (i.e., oral cavity vs. others) had no significant impact on meta-analytic estimates. To further investigate the potential influence of the publication year on the results, we grouped studies into two subgroups: those published before 2015 and those published from 2015 to 2024. This threshold was chosen because digital PET-CT technology began to be widely introduced around 2015. We then compared the pooled sensitivity and specificity between these two subgroups using a chi-squared test. The analysis revealed no statistically significant differences between the groups.

Only Kanamura et al. [33], Zhang et al. [28], and Madsen et al. [35] mentioned contrast injection in their CT protocol (n= 222 patients), so the sample size was too small to investigate the impact of contrast agent injection on the diagnostic performance of PET-CT.

Omitting the per lymph node analysis in the study of Piao et al. [21] did not change the above-mentioned results.

#### 3.1.6. Comparison of the Diagnostic Performance of FDG PET-CT Depending on Different Reference Standards

In this univariate model (forest plot), the standard references are used as denominators.

**Figure 5:**
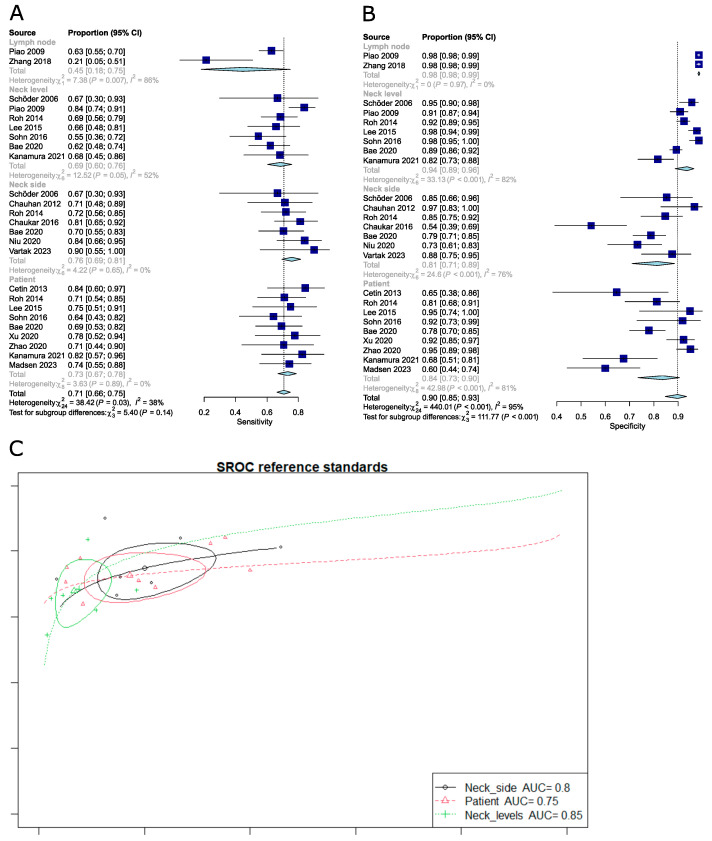
(**A**) Forest plot of sensitivity for different reference standards. (**B**) Forest plot of specificity for different reference standards. (**C**) SROC curve for different reference standards [20,21,22,23,24,25,26,27,28,29,30,31,32,33,34,35].

The pooled sensitivity and specificity for studies analyzed according to different reference standards were as follows: per lymph node 0.45 (95%CI: 0.18–0.75) and 0.98 (95%CI: 0.98–0.99); per neck level 0.68 (95%CI: 0.61–0.75) and 0.93 (95%CI: 0.89–0.96); per neck side 0.76 (95%CI: 0.69–0.81) and 0.81 (95%CI: 0.71–0.89); per patient 0.74 (95%CI: 0.68–0.80) and 0.83 (95%CI: 0.71–0.90). It should be noted that the subgroup analysis for sensitivity was no longer significant using the meta R package (Figure 5); contrary to the Reitsma model bivariate approach, which accounts for both specificity and sensitivity, those are univariate results (only sensitivity). Moreover, when omitting the Piao et al. study [21], subgroup differences appeared to be significant again (Q-test, *p* = 0.002).

#### 3.1.7. Pooled Analysis of the Diagnostic Performance of PET-CT for the Detection of Occult Lymph Node Metastasis (OLNM) in the Early-Stage Disease Subgroup

**Figure 6:**
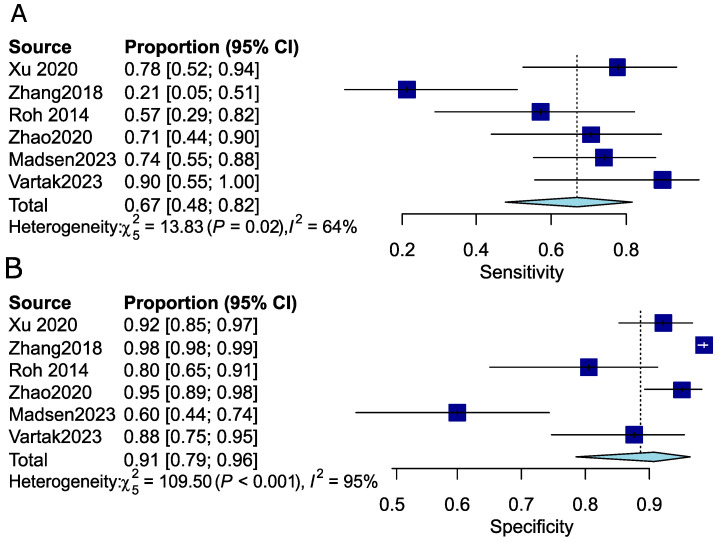
Pooled analysis in the early-stage disease subgroup. (**A**) Forest plot of sensitivity. (**B**) Forest plot of specificity [24,28,31,32,34,35].

Given that OLNM (occult lymph node metastasis) is particularly significant in early-stage disease, we chose to investigate the diagnostic performance of FDG PET-CT specifically in patients with cT1 and cT2 cN0 disease. We identified six studies that either involved patients with early-stage disease or provided data relevant to this stage, comprising 469 patients (Table 1). Prevalence of OLMNM could only be computed for the subgroup of studies using the patient as a denominator. In this subgroup, the prevalence of OLNM was 14.76%. The pooled sensitivity, specificity, and diagnostic odds ratio (DOR) were 0.67 (95%CI: 0.48–0.82), 0.91 (95%CI: 0.79–0.96), and 16.60 (95%CI: 5.02–54.90), respectively (Table 1). The NPV was 0.95 (95%CI: 0.88–0.98). Heterogeneity between studies was high. The I^2^ statistic for sensitivity, specificity, DOR, and NPV was 0.64 (95%CI: 0.13–0.85), 0.95 (95%CI: 0.93–0.97), 0.65 (95%CI: 0.18–0.86), and 0.92 (95%CI: 0.85–0.96), respectively. Forest plots are provided for sensitivity and specificity (Figure 6). Given the low number of studies included in this subgroup analysis, meta-regression was not performed due to the limited statistical power and the risk of overfitting. Further stratification of the meta-analysis according to subsite, tumor grade, or adverse features was not possible as complete data for these subgroups could not be extracted from the included studies.

## 4. Discussion

In this meta-analysis, the diagnostic performance of FDG PET-CT to detect occult lymph node metastases (OLNMs) was assessed based on the available FDG PET-CT literature since the year 2000. Contrary to previous meta-analyses, PET examinations were not included in our analysis as PET scans have been widely replaced nowadays by PET-CT. According to our results, the pooled sensitivity, specificity, diagnostic odds ratios, and negative predictive value (NPV) were 0.71 (95%CI: 0.66–0.75), 0.90 (95%CI: 0.84–0.93), 20.03 (95%CI: 13.51–29.70), and 0.92 (95%CI: 0.89–0.95), respectively. The different reference standards (histology per patient, per neck side, per neck level, or per lymph node) used in the studies included in this meta-analysis were the main cause of inter-study heterogeneity. In the cN0 neck defined based on combined clinical and cross-sectional imaging assessment, the overall FDG PET-CT sensitivity was 0.76 (95%CI: 0.68–0.82), while the specificity and NPV were 0.85 (95%CI: 0.75–0.91) and 0.93 (95%CI: 0.89–0.96), respectively. The diagnostic performance of FDG PET-CT in the cN0 neck was highest in the per neck side analysis following an upward trend from “per lymph node” to “per patient” analysis.

Lymph node metastasis is one of the most accurate predictors of cancer-related outcomes in HNSCC [36]. As shown in the seminal paper of D’Cruz et al. [4], elective neck dissection is the treatment of choice for HNSCC patients with a cN0 neck because of a clear survival advantage over watchful waiting. Although neck dissection has been shown to improve survival, it is an invasive and ultimately sometimes unnecessary procedure for the majority of patients with cN0 status [37], as up to 70–80% of patients who undergo elective neck dissection do not ultimately have nodal disease on final pathology [38]. Following the study of D’Cruz et al., there has been a rise in elective neck dissections in patients with early-stage oral SCC [4].

Several trials demonstrated the feasibility and high diagnostic performance of SLNB with a negative predictive value of up to 96% [6] and the oncologic equivalence of the SNLB and ND approach, nevertheless with better functional outcomes with SLNB [5]. Unfortunately, SLNB remains a time-consuming procedure that does not prevent an open approach and is only available in some centers. Therefore, the identification of a reliable, non-invasive method that is sensitive enough to confirm that the risk of occult lymph node metastases (LNMs) in the neck is below the 20% probability threshold, which is considered the criterion for elective neck dissection, would have a significant impact on patient management. FDG PET-CT has become an important imaging modality to detect LNMs because it provides both morphological and metabolic information [39]. It could then be of interest to explore this as a non-invasive decision-making tool for determining the necessity of elective neck dissections in cN0 neck cases. Several factors can influence the accuracy of PET-CT in detecting occult neck metastases. These include changes in physiologic uptake, inflammatory uptake, primary tumor size, localization, or postsurgical modifications [40,41]. In addition, technical factors such as the timing of the scan, the dose of the radiotracer used, spatial resolution, and interpretation criteria can affect the accuracy of PET-CT. The limitations of this imaging technique are the presence of false positives caused by inflammatory nodes, as well as small or hypometabolic lesions [42]. It is, nevertheless, worth mentioning that FDG PET-CT has a very high diagnostic performance for detecting distant metastases and distant second primary cancers in HNSCC patients, its diagnostic performance being similar to that of more sophisticated hybrid imaging techniques, namely PET/MRI [43].

In 2008, Kyzas et al. [44] performed a meta-analysis to assess the diagnostic performance of FDG PET (no PET-CT examinations included) without discriminating between clinically positive (N+) and clinically negative (N0) necks. The authors analyzed 32 studies, including 1236 patients. They performed subgroup analysis showing that FDG-PET sensitivity dropped from 79% to 50% when analyzing N0 necks, with no specificity difference (86% vs 87%).

In 2012, Liao et al. [45] compared the diagnostic performance of PET scans (without distinguishing between PET and PET-CT) to other imaging modalities and showed that PET was not superior to CT, MRI, or US in the N0 neck workup. Their results showed a pooled sensitivity of 0.66 (95%CI: 0.47–0.80), 0.52 (95%CI: 0.39–0.65), 0.65 (95%CI: 0.34–0.87), and 0.66 (95%CI: 0.54–0.77), and a specificity of 0.87 (95%CI: 0.77–0.93), 0.93 (95%CI: 0.87–0.97), 0.81 (95%CI: 0.64–0.91), and 0.78 (95%CI: 0.71–0.83), for PET, CT, MRI, and US, respectively. Interestingly, they found a similar diagnostic accuracy of PET and US, with a better ability for PET to detect lymph nodes in the retropharyngeal or deep neck spaces.

In 2019, Kim et al. [46] performed a meta-analysis to assess the diagnostic performance of FDG PET or PET-CT (without distinguishing between the two); the authors analyzed 18 studies, including 1044 patients, and found a pooled sensitivity of 58% and a pooled specificity of 87% in the per patient-based analysis. They also found that the test’s sensitivity dropped to 53% in the neck level-based analysis while the corresponding specificity increased to 97%. However, contrary to the results shown in this study, they could not determine that this was a cause of inter-study heterogeneity.

From the present analysis, PET-CT was found to have a higher pooled specificity than other comparative meta-analyses (90% vs. 87% for Liao et al. and Kim et al. [46] or 86% for Kyzas et al. [44]). We also found that using different reference standards among studies was associated with differences in the reported diagnostic performance of FDG PET-CT across studies. When neck levels were taken as the reference standard, specificity was highest. Specificity was also high regarding lymph node analysis, but the power of this two-study analysis is not strong. When the neck side was the reference standard, sensitivity was highest.

Our study found a pooled sensitivity of 71%, which is higher than the pooled sensitivity reported in two [45,46] of the three meta-analyses mentioned above; one possible explanation is that previous meta-analyses included both PET and PET-CT examinations without distinguishing between the two examination types [45]. Nowadays, PET has been widely replaced by PET-CT because the latter has the advantage of precise lesion localization due to fusion of metabolic and morphologic information. Furthermore, administering contrast enhancement offers the possibility to better characterize lymph nodes. Nevertheless, only four of the sixteen studies included in this meta-analysis indicated the use of contrast-enhanced CT images. Due to the limited number of included patients in these four studies, no subgroup analysis could be performed. Our study equally included more recent research and more prospective studies than previous meta-analyses.

The NPV of FDG PET-CT was 92%, indicating that a patient with a cN0 neck as evaluated by FDG PET-CT has only an 8% chance of being pN+ upon pathological analysis. However, the false negative rate (FNR) is more critical in this context, as it measures the test’s ability to detect positive cases, which is essential for identifying cN0/pN+ individuals. The overall FNR was 29%, meaning that FDG PET-CT missed nearly a third of cN0 patients who were found to be pN+ upon pathological analysis. Importantly, in the T1-T2 subgroup of patients, sensitivity of FDG PET-CT was even lower, indicating a pooled FNR of 33%. This high FNR suggests that FDG PET-CT alone is insufficient as a decision-making tool for elective neck dissection.

Furthermore, we know that HNSCC has a different metastatization pattern depending on the location of the primary tumor. However, this meta-analysis included all patients with HNSCC, including patients with glottic tumors who metastasize late to lymph nodes.

In 2023, Madsen et al. [35] aimed to clarify the role of PET-CT in patients with early-stage oral cavity SCC (T1–T2) and cN0 necks. They found that PET-CT was significantly more sensitive than neck MRI (74% vs. 27%, *p* = 0.0001) for N-staging, with a surprising low sensitivity of MRI. The accuracy of PET-CT and neck MRI was comparable, while the NPV was slightly in favor of PET-CT (77% vs. 63%, *p* = 0.16). They concluded that neither PET-CT nor MRI should stand alone for N-staging T1-T2 oral cavity SCC.

On the other hand, in 2023, Vartak et al. [34] found a FDG PET-CT sensitivity of 90%, specificity of 87.5%, and NPV of 97.7%. They concluded that if a decision regarding the need for neck dissection had been based on FDG PET-CT only, the number of neck dissections would have been reduced by 74.13%. We certainly need more studies to reconcile these contradictory data.

These data also highlight the challenges encountered with defining criteria for radiologically identifying pathological lymph nodes [47] and underscore the potential future applications of radiomics [48] and multiparametric PET/MRI [49]. Therefore, future studies may reveal an improved detection of OLNMs in the cN0 neck.

## 5. Conclusions

The specificity and the NPV of PET-CT for occult nodal metastases from HNSCC in patients with a cN0 neck is high. However, the false negative rate, irrespective of the standard reference used (per patient, per neck side, or per neck node level), is too high for deciding whether to perform neck dissection. Further studies are needed to determine the usefulness of FDG PET-CT in patients with HNSCC and cN0 necks, especially in the target population of early oral SCC with a cN0 neck. Additionally, more data are needed about the diagnostic performance of contrast-enhanced FDG PET-CT. The current meta-analysis equally showed that it is of paramount importance to consider the reference standard as well as the definition of a cN0 neck when evaluating the diagnostic performance of PET-CT.

## Figures and Tables

**Table 1:** Study characteristics: Characteristics of studies included in the meta-analysis. The gold standard in all studies was histopathology of neck dissection specimens. However, the definition of cN0 varied from one study to another, as indicated in the table.

	Country	Type of Study	Patients (n)	Reference Standard	cN0 Definition	TP	TN	FP	FN	Sensitivity (%)	Specificity (%)	Accuracy (%)	PPV (%)	NPV (%)	T stages Included	Histological Analysis	PET/CT Indicating Positive Nodes
Schöder 2006 [20]	United states	Prospective	31	Neck level (142)	Clinical + CT/MRI/US	6	127	6	3	67	95	94	50	98	T1/T2/T3/T4	Serial analysis, H&E staining	Non-quantitative, visual focal uptake > background
				Neck side (36)	Clinical + CT/MRI/US	6	23	4	3	67	85	80	60	88			
Piao 2009 [21]	Japan	Retrospective	56	Neck level (345)	Neck palpation	71	236	24	14	83.5	90.8	89	74.7	94.4	Not mentionned	Not mentionned	SUV > 2.5
				Lymph nodes (2705)	Neck palpation	103	2501	40	61	62.8	98.4	96.3	72	97.6			
Chauhan 2012 [22]	India	Prospective	49	Neck side (51)	Neck palpation	15	29	1	6	71.4	96.7	86.3	93.5	82.9	T1/T2/T3/T4	Standard analysis H&E staining	Non-quantitative, visual focal uptake > background
Cetin 2013 [23]	Turkey	Retrospective	36	Patient	Clinical + CT/MRI/US	16	11	6	3	84.2	76.5	75	72.7	78.6	T1/T2/T3/T4	Standard analysis H&E staining	Non-quantitative, visual focal uptake > background
Roh 2014 [24]	Korea	Prospective	91	Patient	Neck palpation	27	43	10	11	71	81	77	73	80	T1/T2/T3/T4	Standard analysis H&E staining	Non-quantitative, visual focal uptake > background
				Neck side (121)	Neck palpation	31	66	12	12	72	85	80	72	85			
				Neck level (466)	Neck palpation	48	366	30	22	69	92	89	62	94			
Lee 2015 [25]	Korea	Retrospective	39	Patient	Neck palpation	15	18	1	5	75	94.7	84.6	93.8	78.3	T1/T2/T3/T4	Standard analysis H&E staining	SUVmax > 2.5
				Neck level (210)	Neck palpation	23	171	4	12	65.7	97.7	92.4	85.2	93.4			
Sohn 2016 [26]	Korea	Retrospective	49	Patient	Neck palpation	16	22	2	9	64	91.7	77.6	88.9	71	T1/T2/T3/T4	Standard analysis H&E staining	SUVmax ≥ 2.5
				Neck level (162)	Neck palpation	18	127	2	15	54.6	98.5	89.5	90	89.4			
Chaukar 2016 [27]	India	Prospective	70	Neck side (85)	Neck palpation	30	26	22	7	82	54	66	57	79	Not mentionned	Not mentionned	SUV > 2.5
Zhang 2018 [28]	Canada	Retrospective	32	Lymph nodes	Neck palpation	3	1237	20	11	21.4	98.4	97.6	13	99.1	T1/T2	Not mentionned	SUVmax > 2.5
Niu 2020 [29]	China	Prospective	78	Neck side (98)	Neck palpation	26	49	18	5	83.9	73.1	76.5	59.1	90.7	T1/T2/T3/T4	Serial analysis H&E staining	Non-quantitative with defined criteria *
Bae 2020 [30]	Korea	Prospective	178	Patient	Neck palpation	29	106	30	13	69.1	77.9	75.8	49.2	89.1	T1/T2/T3/T4	Serial analysis H&E staining	Non-quantitative, visual focal uptake > background
				Neck side (199)	Neck palpation	31	122	33	13	70.5	78.7	76.9	48.4	90.4			
				Neck level (678)	Neck palpation	36	553	67	22	62.1	89.2	86.9	35.0	96.2			
Xu 2020 [31]	China	Prospective	120	Patient	Clinical + CT/MRI/US	14	94	8	4	77.8	92.2	90	63.6	95.9	T1/T2	Not mentionned	SUVmax > 2.5
Zhao 2020 [32]	China	Prospective	135	Patient	Clinical + CT/MRI/US	12	112	6	5	70.6	94.9	91.9	66.7	95.7	T1/T2	Not mentionned	SUVmax > 2.5
Kanamura 2021 [33]	Japan	Retrospective	57	Patient	Clinical + CT	14	27	13	3	82.4	67.5	72	51.9	90	Not mentionned	Not mentionned	SUVmax > 2.0 with combined index
				Neck level (141)	Clinical + CT	15	119	22	7	68.2	84.4	82.2	40.5	94.4			
Vartak 2023 [34]	India	Prospective	51	Neck side (58)	Clinical + CT/MRI/US	9	42	6	1	90	87.5	87.9	60	97.7	T1/T2	Serial H&E staining	SUV max calculated by the inbuilt software of the PET
Madsen 2023 [35]	Denmark	Prospective	76	Patient	Clinical + US+/- FNAC	23	27	18	8	74	60	66	56	77	T1/T2	Not mentionned	Subjective

TP: true positive; TN: true negative; FP: false positive; FN: false negative; PPV: positive predictive value; NPV: negative predictive value; H&E: hematoxylin–eosin; * (1) SUVmax of lymph nodes was higher than background tissue. (2) The long axial diameters of lymph nodes were more than 15 mm if they were in level I or level II, and more than 10 mm if they were in level III to V. (3). Any nodes that were spherical or exhibited edge enhancement, central necrosis, cystic degeneration, or clustered features.

**Table 2:** Effects of moderators.

Variables	Diagnostic Estimate	Regression	SE	z	*p*	95%CI	
Type of study (Retrospective vs. Prospective)	Sensitivity	0.001	0.201	0.007	0.995	−0.392	0.395
	False positive rate	−0.924	0.464	−1.993	0.046	−1.834	−0.015
Sample size	Sensitivity	−0.001	0.002	−0.613	0.540	−0.005	0.003
	False positive rate	0.007	0.005	1.285	0.199	−0.003	0.017
Definition of cN0 (clinico-radiological vs. Clinical)	Sensitivity	−0.232	0.232	−0.999	0.318	−0.687	0.223
	False positive rate	−0.644	0.494	−1.303	0.193	−1.614	0.325
Year	Sensitivity	−0.021	0.023	−0.899	0.369	−0.066	0.025
	False positive rate	0.089	0.047	1.902	0.057	−0.003	0.180
Histological processing (Routine vs. Serial)	Sensitivity	0.18	0.334	0.54	0.589	-0.474	0.835
	False positive rate	−0.09	0.669	−0.134	0.893	−1.401	1.221
Reference standard (Neck Level vs. Neck Side and Whole neck)	Sensitivity	0.485	0.325	1.491	0.136	−0.152	1.122
	**False positive rate**	**1.511**	**0.629**	**2.402**	**0.016**	**0.278**	**2.744**
**Reference standard (Neck Side vs. Neck Level and Patient)**	**Sensitivity**	**0.833**	**0.345**	**2.412**	**0.016**	**0.156**	**1.510**
	**False positive rate**	**2.725**	**0.635**	**4.288**	**0.0000**	**1.479**	**3.970**
**Reference standard (Patient vs. Neck Level and Neck Side)**	**Sensitivity**	**0.715**	**0.331**	**2.158**	**0.031**	**0.066**	**1.365**
	False positive rate	2.590	0.619	4.182	0.0000	1.376	3.804
Localization (Other vs. Oral cavity)	Sensitivity	0.105	0.208	0.504	0.614	−0.303	0.513
	False positive rate	−0.813	0.473	−1.72	0.085	−1.74	0.113
Contrast (Non-injected vs. Injected)	Sensitivity	0.318	0.311	1.023	0.306	−0.291	0.928
	False positive rate	−0.493	0.647	−0.762	0.446	−1.762	0.775

*p*: *p*-value of diagnostic bivariate random effect meta-regression using restricted maximum likelihood (REML) of between-study variance. SE: standard error; z: z-value; CI: confidence intervals.

## Data Availability

The data presented in this study are available in this article.

## Appendix A

### PubMed Database Research Equation 8 July 2024

“Squamous Cell Carcinoma of Head and Neck”[MeSH Terms] OR “Gingival Neoplasms”[MeSH Terms] OR “Palatal Neoplasms”[MeSH Terms] OR “Tongue Neoplasms”[MeSH Terms] OR “Laryngeal Neoplasms”[MeSH Terms] OR “Pharyngeal Neoplasms”[MeSH Terms] OR “Laryngeal Neoplasms”[MeSH Terms] OR “HNSCC”[Title/Abstract] OR “Squamous Cell Carcinoma of Head and Neck”[Title/Abstract] OR “Head And Neck Squamous Cell Carcinoma*”[Title/Abstract] OR “Squamous Cell Carcinoma of the Larynx”[Title/Abstract] OR “Laryngeal Squamous Cell Carcinoma*”[Title/Abstract] OR “Oral Tongue Squamous Cell Carcinoma*” [Title/Abstract] OR “Hypopharyngeal Squamous Cell Carcinoma*”[Title/Abstract] OR “Oral Squamous Cell Carcinoma*”[Title/Abstract] OR “Oral Cavity Squamous Cell Carcinoma*”[Title/Abstract] OR “Oropharyngeal Squamous Cell Carcinoma*”[Title/Abstract] OR “Gingival Neoplasms”[Title/Abstract] OR “Gingival cancer”[Title/Abstract] OR “Palatal Neoplasms”[Title/Abstract] OR “Palatal cancer”[Title/Abstract] OR “Tongue Neoplasms”[Title/Abstract] OR “Tongue cancer”[Title/Abstract] OR “Laryngeal Neoplasms”[Title/Abstract] OR “Larynx Neoplasm*”[Title/Abstract] OR “Cancer of Larynx”[Title/Abstract] OR “Larynx Cancer*”[Title/Abstract] OR “Laryngeal Cancer*”[Title/Abstract] OR “Pharyngeal Neoplasms”[Title/Abstract] OR “Pharynx Neoplasm*”[Title/Abstract] OR “Cancer of Pharynx”[Title/Abstract] OR “Pharynx Cancer*”[Title/Abstract] OR “Pharyngeal Cancer*”[Title/Abstract] OR “Oropharynx Neoplasm*”[Title/Abstract] OR “Cancer of oropharynx”[Title/Abstract] OR “Oropharynx Cancer*”[Title/Abstract] OR “Oropharyngeal Cancer*”[Title/Abstract] OR ”Cancer of hypopharynx“[Title/Abstract] OR “Hypopharynx cancer*”[Title/Abstract] OR “Hypopharyngeal cancer*”[Title/Abstract] AND “Lymphatic Metastasis“[MeSH] OR “Lymphadenopathy“[MeSH] OR “Lymphatic metastasis»[Title/Abstract] OR “Lymph node metastasis»[Title/Abstract] OR “Lymph node metastase*»[Title/Abstract] OR “Nodal metastasis»[Title/Abstract] OR “Metastatic lymph node»[Title/Abstract] OR “Lymphadenopathy”[Title/Abstract] OR “Malignant lymph node”[Title/Abstract] OR “Cervical metastasis”[Title/Abstract] OR “Local metastasis”[Title/Abstract] OR “Adenopathy”[Title/Abstract] OR “Adenopathies”[Title/Abstract] OR “Lymphadenopathies”[Title/Abstract] AND “Positron-Emission Tomography”[MeSH] OR “Positron-Emission Tomography”[Title/Abstract] OR “Positron-Emission Tomography Imaging”[Title/Abstract] OR “PET Scan”[Title/Abstract] OR “18F-FDG-PET-CT”[Title/Abstract] OR “PET-CT”[Title/Abstract] OR “PET Imaging”[Title/Abstract] OR “FDG-PET”[Title/Abstract] OR “Fluorodeoxyglucose-Positron Emission Tomography”[Title/Abstract]

## Appendix B

### EMBASE Database Research Equation 8 July 2024

(‘head and neck squamous cell carcinoma’/exp OR ‘head and neck squamous cell carcinoma’ OR ‘tongue tumor’/exp OR ‘tongue tumor’ OR ‘larynx tumor’/exp OR ‘larynx tumor’ OR ‘pharynx cancer’/exp OR ‘pharynx cancer’ OR ‘oropharynx tumor’/exp OR ‘oropharynx tumor’ OR ‘hypopharynx tumor’/exp OR ‘hypopharynx tumor’ OR ((cancer NEAR/2 (hypopharynx OR larynx OR oropharynx OR pharynx)):ab,ti) OR hnscc:ab,ti OR ((hypopharyngeal NEAR/2 (cancer* OR neoplams OR ‘squamous cell carcinoma*’)):ab,ti) OR ((laryngeal NEAR/2 (cancer* OR neoplam* OR ‘squamous cell carcinoma’)):ab,ti) OR ((oral NEAR/2 (‘cavity squamous cell carcinoma*’ OR ‘squamous cell carcinoma*’ OR ‘tongue squamous cell carcinoma*’)):ab,ti) OR ((oropharyngeal NEAR/2 (neoplasm* OR ‘squamous cell carcinoma*’)):ab,ti) OR ((‘squamous cell carcinoma’ NEAR/2 (‘head and neck’ OR larynx)):ab,ti) OR (((gingival* OR hypopharynx OR larynx OR oropharyngeal OR palatal OR pharyngeal OR pharynx OR tongue) NEAR/2 (cancer OR neoplasm*)):ab,ti)) AND (‘lymph node metastasis’/exp OR ‘lymph node metastasis’ OR ‘cervical lymph node metastasis’/exp OR ‘cervical lymph node metastasis’ OR ‘lymphadenopathy’/exp OR ‘lymphadenopathy’ OR adenopathies:ab,ti OR adenopathy:ab,ti OR ((metastas* NEAR/2 (cervical OR local OR ‘lymph node*’ OR lymphatic OR nodal)):ab,ti) OR lymphadenopathies:ab,ti OR lymphadenopathy:ab,ti OR ((‘lymph node*’ NEAR/2 (malignant* OR metastatic*)):ab,ti)) AND (‘positron emission tomography’/exp OR ‘pet scanner’/exp OR ‘positron emission tomography’:ti,ab OR ‘pet scanner’:ti,ab OR ‘18-fdg-pet*’:ti,ab OR ‘18-fdg-pet-ct’:ti,ab OR ‘positron-emission tomography imag*’:ti,ab OR ‘positron emission tomography’:ti,ab OR ‘pet scan’:ti,ab OR ‘pet-ct’:ti,ab OR ‘pet imaging’:ti,ab OR ‘fdg-pet’:ti,ab OR ‘fluorodeoxyglucose-positron emission tomography’:ti,ab)
